# Supplementary material for: MetaRibo-Seq measures translation in microbiomes
Source: Nat Commun. 2020 Jun 29;11:3268. doi: 10.1038/s41467-020-17081-z (PMC7324362; doi:10.1038/s41467-020-17081-z)
Supplement: Supplementary file 10 — Supplementary Data 7 [file 41467_2020_17081_MOESM10_ESM.zip › File2/Confidence_High_Taxonomy/93306_out.krona.html]

Javascript must be enabled to view this page.

members
magnitude
magnitudeUnassigned
count
unassigned
taxon
rank

93306\_out

6

superkingdom
6
2

1224
1
phylum

1
class
1236

order
1
72274

1
family
468

genus
1
475

1
species

SRS893270\_contig\_number\_7279
90239

1239
phylum
5

186801
5
class

186802
order
5

5
family
541000

5
species

SRS043768\_contig\_number\_23653SRS045244\_contig\_number\_3078SRS1055043\_contig\_number\_19143SRS142599\_contig\_number\_25939SRS143991\_contig\_number\_103
1952288
